# The complete mitochondrial genome of the Mexican blind brotula *Typhlias pearsei* (Ophidiiformes: Dinematichthydae): an endemic and troglomorphic cavefish from the Yucatán Peninsula karst aquifer

**DOI:** 10.1080/23802359.2022.2087558

**Published:** 2022-06-24

**Authors:** Jairo Arroyave, Adán Fernando Mar‐Silva, Píndaro Díaz-Jaimes

**Affiliations:** a Instituto de Biología, Universidad Nacional Autónoma de México, Ciudad de México, México; b Laboratorio de Genética de Organismos Acuáticos, Instituto de Ciencias del Mar y Limnología, Universidad Nacional Autónoma de México, Ciudad de México, México; c Posgrado en Ciencias del Mar y Limnología, Universidad Nacional Autónoma de México, Ciudad de México, México

**Keywords:** troglobitic fish, viviparous brotula, Bythitoidei, ophidiiforms, mitogenomics

## Abstract

In this study we report the first complete and annotated mitochondrial genome of the Mexican blind brotula, *Typhlias pearsei*, a troglobitic cavefish endemic to the Yucatán peninsula karst aquifer in southeastern Mexico. Genomic sequencing was accomplished via next generation sequencing (NGS). The resulting mitogenome is 16,813 bp long and, as in most vertebrates, consists of a total of 37 genes (13 PCGs, 2 rRNAs, 22 tRNAs) and two non-coding regions (control region and origin of the light strand replication). Other than a rearrangement in the position of two tRNAs (shuffling between tRNA-Ile and tRNA-Gln), the mitogenome of *T. pearsei* exhibits a genomic composition and organization similar to that of most teleost mitogenomes. Besides offering this valuable genomic resource for future studies, the resulting mitogenome was used in a comparative context to test the current higher-level taxonomy of ophidiiform fishes and to examine the phylogenetic position of *T. pearsei* among viviparous brotulas. Our phylogenetic results confirm those from the most comprehensive molecular phylogenetic study of the group.

*Typhlias pearsei* (Hubbs 1938) is a troglobitic viviparous brotula endemic to the Yucatán Peninsula karstic aquifer (Møller et al. 2016; Scharpf 2017; Arroyave 2020). The species appears to be confined to habitats out of reach of sunlight, for besides submerged caves it is only known to inhabit cenotes (natural water-filled sinkholes) located inside dry caves (Arroyave et al. 2019). Its cave-dwelling nature, however, has made it very challenging to sample and study it, and therefore very little is known about its biology, including basic information on its genetic background and variation. Hence the need and importance of generating for the first time its complete annotated mitochondrial genome. Besides offering this valuable genomic resource for future studies, the resulting mitogenome is herein used in a comparative context to test previous hypothesis regarding the phylogenetic position of *T. pearsei* and the systematics of ophidiiform fishes. To date, only one dedicated study has investigated the systematics of ophidiiforms using only one nuclear and two mitochondrial partial gene fragments (Møller et al. 2016). Whereas an overhauling of the classification of the order at the familial level followed from their phylogenetic results, the general conclusions of Møller et al. (2016) have not been tested with independent datasets, let alone at a mitogenomic scale. The mitogenome of *T. pearsei* presented herein constitutes the first available mitogenome from a member of the family Dinematichthydae and adds to a collection of published ophidiiform mitogenomes, thus allowing testing of the higher-level phylogenetic taxonomy offered by Møller et al. (2016), including the phylogenetic placement of *T. pearsei* amongst major ophidiiform linages as well as interfamilial relationships within Ophidiiformes.

The individual of *T. pearsei* used to generate the mitogenome presented here was collected from a submerged cave connected to the cenote Sacahua (21°14′30″N, 89°0′15.80″W) in the municipality of Dzidzantún, state of Yucatán, using a custom-made hand net specifically designed for efficient capture and secure storage while cave diving, and under collecting permit SGPA/DGVS/05375/19 issued by the Mexican Ministry of Environment and Natural Resources to JA. The voucher specimen is deposited in the Colección Nacional de Peces (CNPE) of the Instituto de Biología (IB) at the Universidad Nacional Autónoma de México (UNAM) (http://www.ib.unam.mx/cnpe/; Héctor Espinosa (Curator), hector@unam.mx) under catalog number CNPEIBUNAM 23278. High-molecular genomic DNA was extracted using the phenol-chloroform protocol (Deininger 1990) from a fresh tissue sample (fin clip) taken prior to specimen fixation and preservation. Extracted DNA was sheared by sonication with a Bioruptor pico^®^ of Diagenode and Minichiller^®^. For library preparation we used a DNA sample of 200 ng which was quantified using a Qubit fluorometer (Invitrogen). Library preparation was carried out using the KAPA Biosystem Hyper Kit (Kapa, Biosystem Inc., Wilmington, MA). Fragmented DNA was ligated to custom, TruSeq-style dual-indexing adapters (Glenn et al. 2019). Fragments were size selected in a ∼300–500 bp range which was enriched through PCR, purified and normalized. The Illumina NextSeq v2 300 cycle kit was used for sequencing paired-end 150 nucleotide reads at the Georgia Genomics Facility, University of Georgia, Athens, GA.

The complete mitochondrial genome of *T. pearsei* reported here (OM320983) is 16,813 bp in length and consists of 37 genes (13 PCGs, 2 rRNAs, 22 tRNAs) and two non-coding regions (control region and origin of the light strand replication). Twenty-eight of these 37 genes (12 PCGs, 2 rRNAs, 14 tRNAs) plus the non-coding control region are located on the H-strand, while the remaining nine genes (*NAD6* and 8 tRNAs) are located on the L-strand. The overall base composition of the mitogenome (*A* = 30%, *T* = 24.6%, *G* = 14.4%, and *C* = 30.9%) is biased toward A + T (54.7%) and displays positive AT (0.0996) and negative GC (−0.3646) skewness, a general pattern shared with other ophidiiform fishes (Miya et al. 2003; Fromm et al. 2019; Tabassum et al. 2020). The genomic organization in *T. pearsei* follows that reported for other ophidiiforms and most teleosts (Miya et al. 2003), except for the switching of tRNA-Ile and tRNA-Gln genes, a rare gene rearrangement also documented in *Kurtus gulliveri* (Gobiiformes) (Satoh and Katayama 2022). This rearrangement corresponds to the ‘shuffling’ type, a positional exchange between two mitochondrial genes, and one of the three kinds of mitogenomic rearrangements described for fishes, besides translocation and inversion (Gong et al. 2013).

Phylogenetic relationships were inferred from a data matrix resulting from the concatenated alignment of all 13 PCGs extracted from available annotated ophidiiform mitogenomes, totaling nine ingroup species representing both suborders of the Ophidiiformes (Figure 1). The holocentrid genus *Sargocentron* (*S. rubrum*) was used as outgroup based on a recent higher-level classification of ray-finned fishes (Hughes et al. 2018). Sequences were aligned with MUSCLE (Edgar 2004) and individual alignments were subsequently concatenated, yielding a data matrix totaling 11,457 aligned bp. The best-fit substitution model for each PCG alignment was determined with jModelTest2 (v. 2.1.10) (Darriba et al. 2012). Maximum Likelihood inference of phylogeny was carried out on the concatenated alignment partitioned by gene using RAxML-NG (v. 1.0.1) (Kozlov et al. 2019). The resulting phylogeny (Fig. 1) is consistent with the higher-level systematics of ophidiiform fishes proposed by Møller et al. (2016), thus adding further support to this classification scheme.

**Figure 1.**
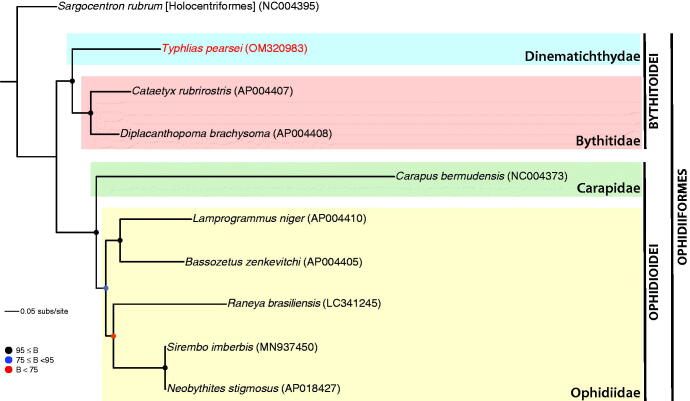
Phylogenetic relationships among select ophidiiform fishes (order Ophidiiformes) inferred via Maximum Likelihood analysis of comparative mitochondrial protein-coding gene sequence data (13 PCGs) sampled from nine ingroup taxa (including the species sequenced in this study, *Typhlias pearsei*, in red font) with representation of both ophidiiform suborders (Bythitoidei and Ophidiodei) and most currently recognized families. Codes in brackets following scientific names correspond to GenBank accessions. Colored circles on nodes indicate degree of clade support as determined by bootstrap (B) values.

## Data Availability

The genome sequence data generated herein that support the findings of this study (complete annotated mitochondrial genome of *Typhlias pearsei*) are openly available in NCBI (https://www.ncbi.nlm.nih.gov) under GenBank accession OM320983. The associated BioProject, SRA, and BioSample numbers are PRJNA822665, SRR18739220, and SAMN27257021, respectively.
